# Dietary crude protein levels during growth phase affects reproductive characteristics but not reproductive efficiency of adult male Japanese quails

**DOI:** 10.5713/ab.21.0060

**Published:** 2021-08-25

**Authors:** Pâmela Lacombe Retes, Danusa Gebin das Neves, Laryssa Fernanda Bernardes, Victoria Veiga Alves, Natália de Castro Gonçalves, Diego de Rezende Lima, Renata Ribeiro Alvarenga, Barbara Azevedo Pereira, Alireza Seidavi, Márcio Gilberto Zangeronimo

**Affiliations:** 1 Department of Veterinary Medicine, Federal University of Lavras, Lavras, MG 37200, Brazil; 2 Department of Animal Science, Federal University of Lavras, Lavras, MG 37200, Brazil; 3 Department of Animal Science, Rasht Branch, Islamic Azad University, Rasht, 4147654919, Iran

**Keywords:** Fertility, Growth Curve, Quail Breeding, Semen Quality, Testicular Development

## Abstract

**Objective:**

The objective was to evaluate the influence of different dietary crude protein (CP) levels during the growth phase on reproductive characteristics and reproductive efficiency as well as the body development of adult male Japanese quail.

**Methods:**

Three hundred one-day-old male quails were distributed into five treatments with diets containing different CP levels (18%, 20%, 22%, 24%, and 26%) in a completely randomized design, with six replicates of ten birds each. The CP diets were applied only during the growth phase (1 to 35 days). At 36 days of age, the birds were transferred to 30 laying cages with three males and nine females each, and all birds received the same diet formulated to meet production-phase requirements until 96 days of age.

**Results:**

The growth rate of the birds increased linearly (p<0.01) with increasing dietary CP, but the age of maximum growth decreased (p<0.05). At growth maturity, all birds had the same body weight (p>0.05). At 35 days of age, higher weight gain was obtained (p<0.05) with diets containing 22% CP or higher. No effects on feed conversion were observed in this phase. The increase in dietary CP enhanced (p<0.01) nitrogen intake and nitrogen excretion but did not affect (p>0.05) nitrogen retention. Testis size, seminiferous tubular area, number of spermatogonia, and germinal epithelial height at 35 days of age increased linearly (p<0.05) with dietary CP, while the number of Leydig cells decreased (p<0.01). The Sertoli cell number at 60 days of age increased linearly (p<0.01) with dietary CP. Dietary CP levels did not affect cloacal gland size, foam weight, foam protein concentration, semen volume, or flock fertility at 90 days of age.

**Conclusion:**

Dietary CP concentration affected body and testicular development in male Japanese quails but did not affect reproductive efficiency.

## INTRODUCTION

Quails are bred worldwide for both meat and egg production because of their ease of handling, small size compared with chickens and broilers, precocious development, low space requirements, low investment requirement, and high egg production capacity and rates. In addition, quail breeding has been used as a good experimental model for bird research because of the birds’ rapid growth, greater egg production, and shorter intervals between generations relative to other species.

Nutrition is a main factor affecting quail productive efficiency. Dietary crude protein (CP) is an important source of amino acids that are used for maintenance, growth, and production demands [1]. During the growth phase, the presence of amino acids is directly related to bird body development [2], and both excesses and deficiencies in amino acids may impair the functions of the organs. Studies have shown that body development is highly correlated with reproductive organ development [3]. Delays in reproductive organ development caused by inadequate dietary CP levels may therefore be directly related to bird sexual maturity and reproductive performance [2].

Regarding quails’ nutritional requirements, most current recommendations [4,5] are based on the performances of commercial unsexed birds or female birds only. However, since males and females present different growth characteristics, their nutritional requirements should also differ. Male and female quails can be bred separately during the growing phase but not during the reproductive phase. To date, there is not enough information to clarify whether males should be fed different diets than females during the growth phase. It is known that in one-day-old chick production systems, most reproductive problems are due to factors related to males because they are present in smaller numbers than females [6]. Thus, different feeding programs considering sex differences could be adopted to increase reproductive indexes [7]. In broiler breeders, studies have shown that separate feeding arrangements for each sex resulted in improved reproductive indexes [3], but in quails, this practice has no proven positive results. Thus, the objective was to evaluate the influences of different dietary CP levels during the growth phase on reproductive characteristics and reproductive efficiency as well as on body development of male Japanese quail.

## MATERIALS AND METHODS

### Animals and experimental design

This experiment was performed between March and June 2018 at the Department of Animal Science of the Federal University of Lavras (UFLA), Lavras, state of Minas Gerais, Brazil. The Animal Research Ethics Committee approved all experimental procedures under protocol no. 057/2017.

The 300 male one-day-old Japanese quails (*Coturnix coturnix japonica*) in the experiment were housed in a masonry shed in 30 cages (50 cm wide×70 cm deep×25.5 cm high) during the growth phase (1 to 35 days). For the production phase (36 to 96 days), the three males with the live weight closest to the cage average were selected, transferred to a screened laying shed, and housed together with nine females in laying cages (32 cm wide×38 cm deep×16 cm high). The temperature and humidity in the sheds were monitored using thermohygrometers placed at bird height, which recorded the minimum and maximum temperature and humidity. The temperature was kept at 38°C during the first three days using a wood-burning stove and was decreased by 0.5°C each day until the birds were 28 days old [8]. The photoperiod was 24 hours light:0 hours dark (natural + artificial light with 60 W lamps) for the first two days of life, 23 hours light:1 hour dark until day 15, then 14 hours light:10 hours dark until the end of the growth phase. At 36 days of age, the light period was increased by 30 min each day up to 17 hours light:7 hours dark, which was retained until the breeding period ended [9].

For both phases (growth and production), a completely randomized design was used, with five treatments and six replicates (cages). The treatments consisted of five dietary CP levels (18%, 20%, 22%, 24%, and 26%) during the growth phase only; feeds were isoenergetic and isonutritious for the remaining nutrients. Diets were formulated based on the recommendations of Rostagno et al [5] by increasing or decreasing the recommended CP level (22% CP) by 2% or 4%. During the production phase, all birds received standard feed (18%) formulated per the recommendations for that phase [5]. All diets were corn and soybean meal-based and formulated using the CP values obtained from their chemical analysis. Feed samples were also collected to determine the CP concentrations (Table 1). Feed and water were supplied *ad libitum* throughout the entire 96-day experimental period.

### Body development and performance

On the first day of the experiment, two previously identified males per cage were individually weighed every three days until 60 days of age to determine the growth curve. The two birds’ live weights were used to calculate the Gompertz curve, which was then used to determine the body weight at the growth maturity (A), growth rate (B), and age of maximum growth (M) [7]. The curve was calculated using Statistica 13.3, using the following model:


$$Wd=A\times e^{\left(-e^{\left(-B\times (d-M)\right)}\right)}$$

in which Wd is the weight at day d (in g); A is the body weight at growth maturity (g); B is the growth rate (d^−1^); and M is the age (d) of maximal growth (inflection point).

The birds were weighed at 1, 14, and 35 days old to determine weight gain. The supplied feed and leftovers were also weighed to determine feed intake. Feed conversion was calculated as the feed intake:weight gain ratio per period.

### Nitrogen balance

At 33, 34, and 35 days of age, the total excreta were collected once daily to evaluate the nitrogen balance at the end of the growth phase. At the beginning and end of the collection period, the feed was weighed to determine intake. Collection trays were inserted under each cage and lined with resistant plastic to avoid contamination and loss of excreta. All collections began at 08:00. Feathers and feed particles were removed, and excreta were placed in plastic bags and stored at −20°C until the end of the collection period. The excreta were then thawed, homogenized, and weighed. Aliquots of 300 g were removed and predried in a forced air circulation oven (55°C) for 72 hours. The samples were again weighed to determine the predry matter and then ground using a knife mill equipped with a 2-mm sieve. Excreta and feed samples were analyzed to determine dry matter and nitrogen concentrations [10]. The nitrogen intake and excreta were quantified based on the results, and the percent nitrogen retained was calculated.

### Testicular development

At 35 days of age, before transfer to the production phase housing, two males from each experimental unit were selected based on their average weight, euthanized by cervical dislocation and exsanguination, and dissected for testicular harvesting. At 60 days of age, the same procedure was performed on one male from each experimental unit. The right and left testes were removed, weighed separately, and measured (width×thickness×height) using a digital pachymeter (Digimess, São Paulo, Brazil). Testicular weight was calculated as the sum of the weight of the right and left testes. Testicular volume was calculated using the equation *V* = 4÷3πab^2^, where *a* is half the testis height and *b* is half the testis width [11]. The gonadosomatic index, which is the percentage of body weight contributed by the gonads, was estimated using the equation *GI* = ([*WTl*+*WTr*]/*LW*)×100, where *WTl* is the left testis weight, *WTr* is the right testis weight, and LW is the live weight. After measuring these parameters, the testes were fixed in Bouin’s solution for approximately 12 hours at ambient temperature and then washed in 70% alcohol for subsequent histological analysis [12].

### Histological analysis

The testes were histologically analyzed at the Laboratory of Histology and Immunohistochemistry of the Department of Animal Science of UFLA. First, the testes were dehydrated in a graded ethanol-xylol series and embedded in paraffin. Sections (5 μm thick) were obtained, suspended in a water bath at approximately 37°C, mounted on silanized histological slides, and dried in an oven at 37°C overnight. The samples were then deparaffinized, rehydrated in a xylol-ethanol series, and stained with hematoxylin-eosin [13].

Seminiferous tubule images were analyzed at 400× magnification using an Olympus CX31 microscope (Olympus, Tokyo, Japan) coupled to an Altra SC30 digital camera (Olympus, Japan) and Axio Vision software (Carl Zeiss, Oberkochen, Germany). In each section, the largest and smallest diameters of ten random round-shaped tubules were measured to calculate the seminiferous tubular area using the equation *A* = *πr*^2^, where *r* is the tubule radius calculated from the average of the largest and smallest diameters. The seminiferous epithelial height was obtained by performing seven measurements per tubule and analyzed to calculate the area.

### Immunohistochemistry

Immunohistochemical analyses were performed in the Laboratory of Histology and Immunohistochemistry of the Department of Animal Science of UFLA. Slides were prepared as described for the histological analyses per Suvarna et al [13] with some adaptations. All slides were incubated in a humidified chamber, and all washes consisted of three consecutive immersions for five minutes in 0.1 M phosphate-buffered saline (PBS) at pH 7.2. Endogenous peroxidase activity was blocked by incubation in Peroxidase Block (K4011, DakoCytomation, USA) for 30 minutes and washed in PBS. Nonspecific antibody binding was blocked by incubating the sections with Block Serum (X0909, DakoCytomation, USA) for 15 minutes. The histological sections were then washed again in PBS, incubated for 2 hours with mouse anti-PCNA monoclonal primary antibody (M0879, DakoCytomation, USA) for staining the Spermatogonia, or incubated for 2 hours with rabbit anti-Androgen receptor polyclonal antibody (AB168834, Abcam, Waltham, MA, USA) for detection of Leydig cells [14]. After incubation the histological sections were washed in PBS, incubated for 15 minutes in Biotinylated Link Universal (K0690 DakoCytomation, USA), washed again in PBS for 5 minutes, and incubated in Streptavidin-HRP (K0690 DakoCytomation, USA) for 15 minutes per the recommendations from the LSAB+System/HRP anti-rabbit and anti-mouse secondary antibody kit (K0690, DakoCytomation, USA). After washing, the histological sections were developed enzymatically using 3,3-diaminobenzidine tetrahydrochloride (DakoCytomation, USA) and immersed in distilled water to stop the reaction after 30 seconds (samples from birds aged 35 days) or after one minute (samples from birds aged 60 days). The slides were then counterstained with hematoxylin and mounted under a coverslip.

**Figure 1:**
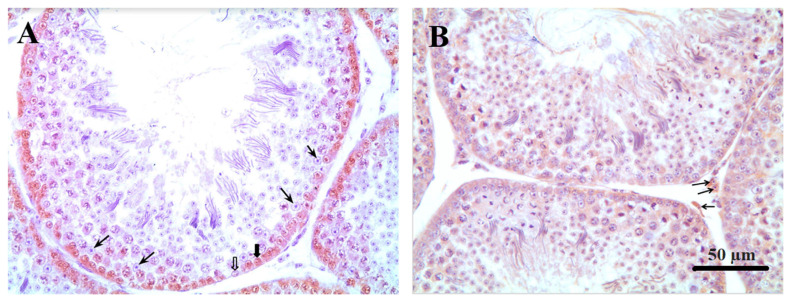
Immunohistochemical localization of spermatogonia; Sertoli and Leydig cells in testicle of Japanese quail at 60 days of age. (A) Thin arrows: Sertoli cells. Thick arrow: stained spermatogonia. Unfilled arrow: unstained spermatogonia. (B) Arrows: Leydig cells (magnification, ×400).

Sertoli cells were identified in counterstained histological section slides of the PCNA test through their morphology. They are tall simple columnar cells, which span from the basement membrane to the lumen surrounded by the proliferating and differentiating germ cells. After counting the number of Sertoli cells per tubule the percentage of proliferating spermatogonia were determined in ten tubule sections per testis and observed at 400× magnification using a light microscope (Olympus CX31; Olympus, Japan). The Sertoli cell number per area (10,000 μm^2^) was calculated using the tubular area. The number and percentage of proliferating spermatogonia were determined by counting the stained cells relative to the total number of cells located closer to the basal membrane (Figure 1).

The number of Leydig cells and the tubular and intertubular areas were evaluated at 1,000× (35 days of age) 400× (60 days of age) using an Olympus CX31 microscope (Olympus, Japan) coupled to an Altra SC30 digital camera (Olympus, Japan) in Axio Vision software (Carl Zeiss, Germany). Ten fields randomly distributed in the testicular parenchyma were evaluated on a projected grid in ImageJ version 1.50i (NIH, Bethesda, MD, USA), and 102 intersection points were considered. The Leydig cell number per area (10,000 μm2) was calculated using the proportion of Leydig cells per intertubular area and the intertubular area.

### Fertility test

On days 59 and 60 of the experiment, all eggs were collected from each cage. Eggs that were cracked, broken, dirty, too large, or too small were discarded, and the remainder (n = 100/treatment) were labeled and stored for 24 hours at 20°C. The eggs were then weighed, and ten eggs were selected per cage based on average weight. The selected eggs were sterilized with a 2:1 formaldehyde (37%):potassium permanganate (99%) solution [15] and incubated at 37.5°C and 60% humidity [16] in an automatic incubator (Luna 480; ChocMaster, Piraquara, Brazil). The automatic egg-turning tray turned the eggs every two hours until day 15 of incubation [17]. After 21 days of incubation, the numbers of hatched and fertilized but unhatched eggs were counted, and percent fertility was calculated.

### Semen quality

Semen quality was evaluated from one male per cage. From day 50 of the experiment, semen was collected every two days via dorsal-abdominal massage.

Semen was evaluated with or without the presence of foam [18] from the two remaining quails in each experimental unit. Foam was collected from each male’s cloacal gland at days 80, 81, and 82 of the experiment at 08:00 and 16:00 each day by gently squeezing the cloacal gland on each side. After collection, the foam was stored in Falcon tubes at −20°C. At the end of the collection period, the samples obtained on the previous days were thawed at ambient temperature and diluted to 1:4 (foam:saline) in saline solution (0.9%). The samples were then centrifuged at 3,000 g for 45 min (Sorvall ST 16 Centrifuge; Thermo Fisher Scientific, Waltham, MA, USA). The supernatant was collected and stored at −80°C until semen quality analysis.

Beginning at 90 days of age, the bird semen was collected three times at 3-day intervals per Burrows and Quinn [19]. Before collection, the feathers in the pericloacal region were removed, and the cloacal gland was measured (width×thickness×height) using a digital pachymeter (Digimess, São Paulo, Brazil) to calculate the cloacal gland area (GA) using the equation GA = *W*×*H*, where W is the lateral width and H is the dorsoventral height [20].

Foam was removed from the cloacal gland during semen collection. The birds were massaged dorsally, beginning near the wing base and ending near the cloaca, with the standard six movements per animal. Slight pressure was then applied with the fingers on the base of the phallus and on the ampoules of the vas deferens to discharge the semen. The seminal content was collected in graduated capillary tubes, where its volume was measured, and then immediately diluted to 1:1 (semen:saline) in saline solution (0.9%). A 1 mL aliquot of solution was diluted with 499 ml of formalin for subsequent sperm concentration analysis, which was performed using a Neubauer chamber. Another 1 mL aliquot was diluted with 98 mL of 0.9% saline solution or 0.9% saline solution + 5% foam [21].

Immediately after dilution in saline solution or saline solution + foam, three trained evaluators blinded to the sample identity evaluated the sperm motility and movement intensity in three subsamples mounted between glass slides and coverslips at 37°C, observing them at 200× magnification using a light microscope (Olympus CX31; Olympus, Japan). Sperm motility was expressed as the percentage of motile spermatozoa, and movement intensity was classified from 0 to 5, with 0 being the lowest and 5 the highest.

Sperm viability was evaluated by mixing one drop of semen with one drop of eosin-nigrosin on glass slides and observing the mixture at 400× magnification using a light microscope (Olympus CX31; Olympus, Japan). The numbers of living (no color) and dead (pink) cells were counted, and sperm viability was calculated as living cells/total cells ×100.

### Statistical analysis

The Gompertz curve parameters (A, B, and M) obtained for each experimental unit and the male reproductive performance and quality data were checked for normality (Anderson-Darling), homoscedasticity (Breusch-Pagan), and independence of error (Durbin-Watson). When the assumptions of normality were met, an analysis of variance (one-way analysis of variance [ANOVA]) was performed, and a regression analysis was performed for the protein levels. Only for live weight a two-way ANOVA (CP levels × age of birds) was performed. When a linear regression could not be fit (R^2^<0.70), a broken-line analysis was performed to determine the best protein level [22]. When a curve could not be fit by broken-line analysis, the averages were compared using the Student-Newman-Keuls (SNK) test at p≤ 0.05. When the ANOVA assumptions were unmet and the Box-Cox and Johnson data transformations could not be used to normalize the data, the data were subjected to a nonparametric analysis, and the averages were compared using the Kruskal-Wallis test. The fetility test was analyzed by a binomial score (1 or 0) based on the presence or absence of fertilization. All statistical analyses were performed using Statistica 13.3 and Action 3.5 software.

## RESULTS

### Growth characteristics and performance

**Figure 2:**
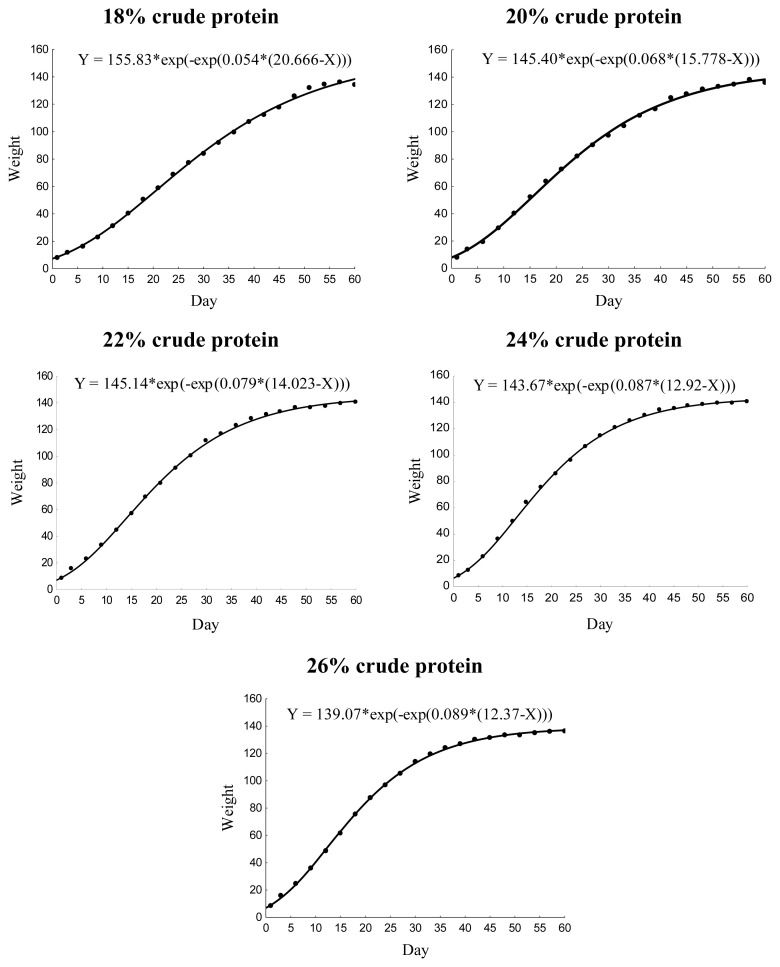
Gompertz model growth curves for male Japanese quails receiving diets with different crude protein levels during the growth phase (n = 12).

**Figure 3:**
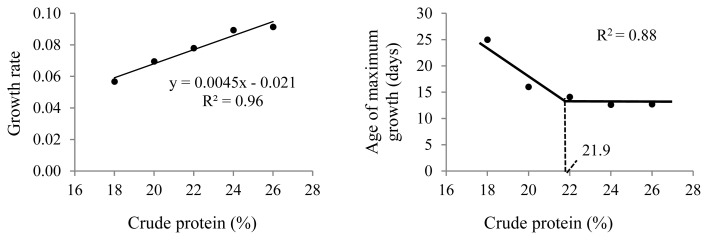
Growth characteristics obtained from the Gompertz curve for male Japanese quails, aged 1 to 60 days, receiving diets with different crude protein levels during the growth phase (n = 12).

The different dietary CP levels in the growth diets resulted in different growth patterns in male quails (Table 2; Figure 2). The growth rate increased linearly (p<0.01), and the age of maximum growth (curve inflection point) decreased (p< 0.05) with increasing dietary CP up to 22% CP (Figure 3). No effect was observed on body weight at maturity (p>0.05).

**Figure 4:**
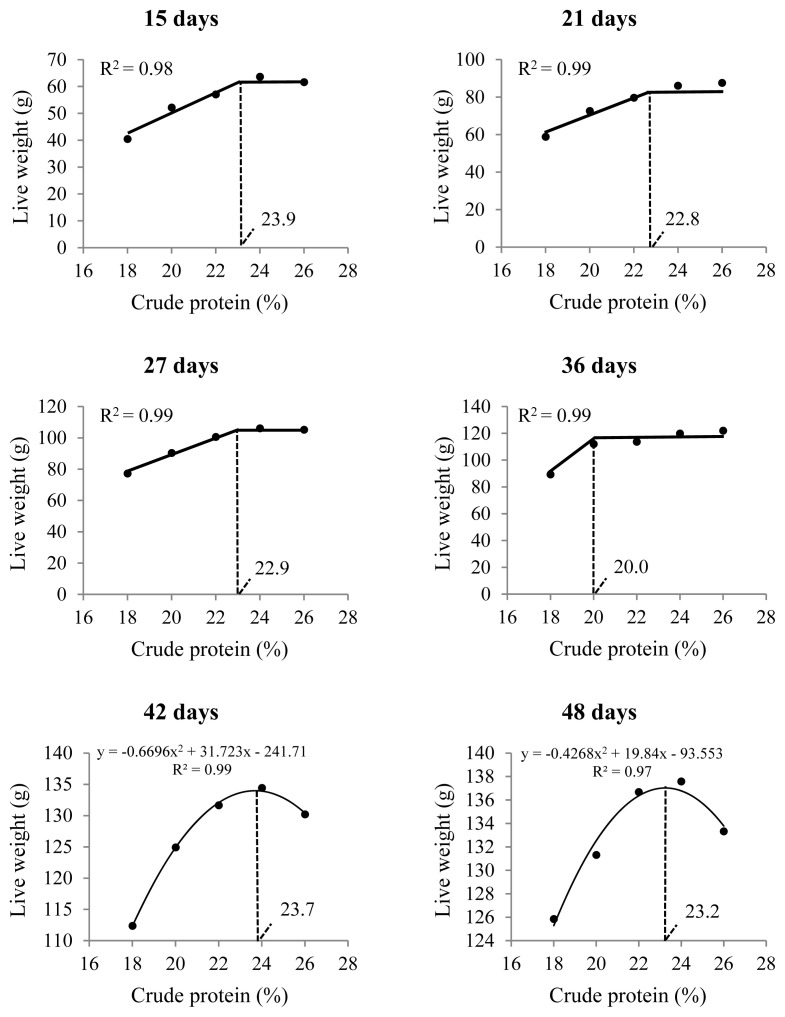
Live weight of male Japanese quails, at different ages, receiving diets with different crude protein levels during the growth phase (n = 60).

Live weight from the second to the seventh week of the birds’ lives was affected by the inclusion of CP (Table 3); 23% CP or higher resulted in greater live weights up to 36 days of age (Figure 4). To obtain greater weights at 42 and 48 days of age, 23.5% CP is recommended.

Diets with 24% and 26% CP resulted in greater weight gain and improved feed conversion up to 14 days of age (p<0.01) (Table 4). Feed intake was lower in the 18% CP group (p< 0.01). Considering the whole growth phase (1 to 35 days), diets with 22% CP or more resulted in less weight gain (p< 0.01). No effect on feed conversion was observed in this phase (p>0.05). At the end of the growth phase (33 to 35 days of age), both nitrogen intake and nitrogen excretion increased linearly with increasing dietary CP (p<0.01). No differences in nitrogen retention were observed (p>0.05).

### Reproductive characteristics and reproductive efficiency

**Figure 5:**
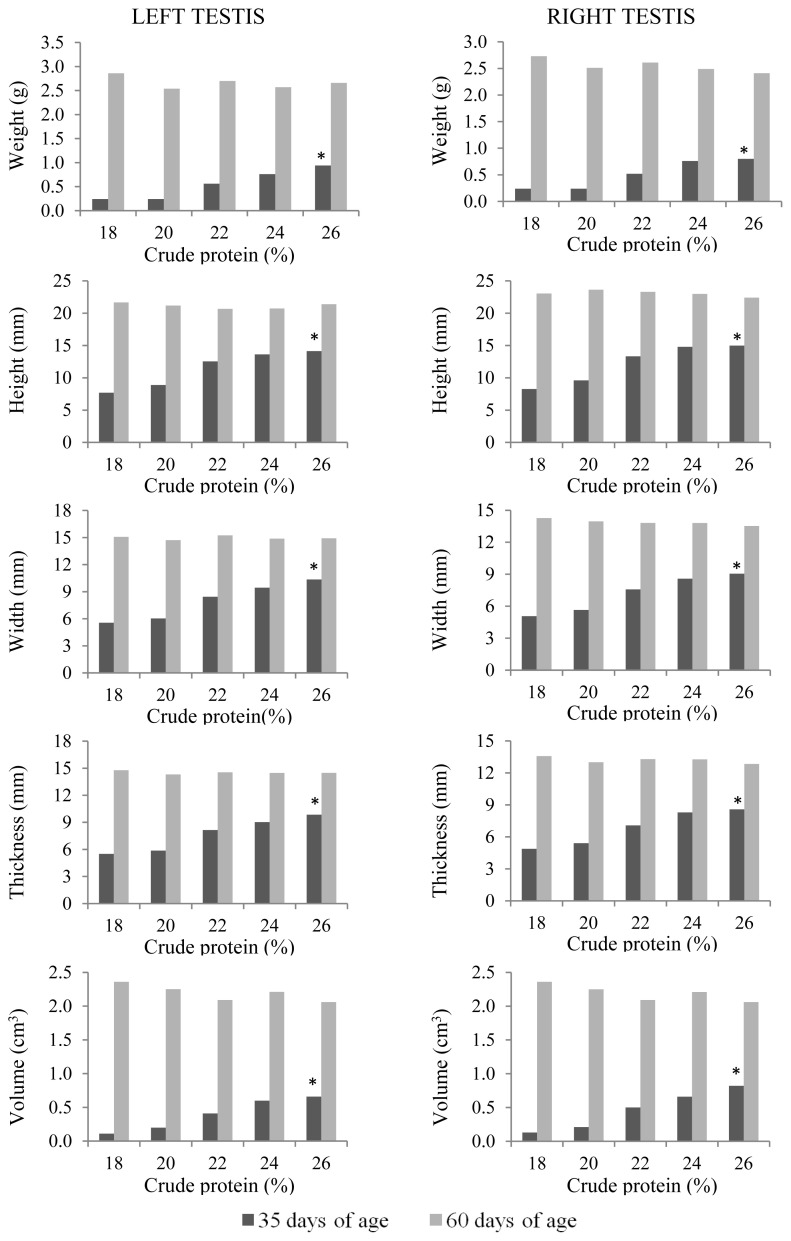
Anatomical characteristics of the left and right testes of male Japanese quails, aged 35 (n = 12) and 60 days (n = 6), receiving diets with different crude protein levels during the growth phase. * Linear effect (p<0.01).

**Figure 6:**
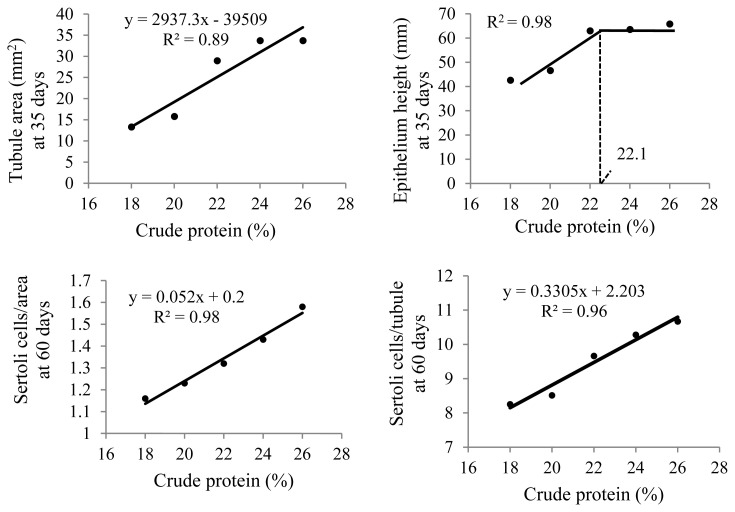
Seminiferous tubular area and testis germinal epithelial height at 35 days of age (n = 12) and number of Sertoli cells per area and per tubule at 60 days of age (n = 6) in Japanese quails receiving diets with different crude protein levels during the growing phase.

At 35 days of age, anatomical and histological evaluations of the quail testes were affected by the dietary CP level (p<0.01) (Table 5). The sizes (height, width, and thickness), as well as weight and volume, of the right and left testes and seminiferous tubular areas increased linearly (p<0.01) with increasing dietary CP levels (Figure 5). Germinal epithelial height increased (p<0.01) only up to 22% CP (Figure 6). A higher intertubule: tubule ratio was observed (p<0.01) in the 24% and 26% CP groups. There was a linear effect (p<0.01) of dietary CP on the number of spermatogonia and Leydig cells in the testis.

At 60 days of age, a linear increase (p<0.01) in Sertoli cell numbers was observed with increasing CP levels in the diet. Higher Leydig cell numbers were observed (p<0.01) with 18% CP and more spermatogonia/area were observed with 24% and 26% CP (p<0.05).

At 90 days of age, the CP levels in the growth diet did not affect cloacal gland size, foam weight and protein concentration, semen volume and quality, or flock fertility (p>0.05) (Table 6).

## DISCUSSION

Although dietary CP levels affected body development, Sertoli cell numbers, and the number of spermatogonia in male Japanese quails, their reproductive efficiency during the production phase was unaffected. These results suggest that the lowest CP level tested (18% CP) or the CP level recommended for females (24%) [4,23] can be used in breeder flocks.

Male fertility is essential to one-day-old chick production systems and is directly related to spermatogenesis. Spermatozoid production, in turn, depends on the Sertoli cell number and activity and the number of proliferating spermatogonia [24]. In the present study, the increased Sertoli cell numbers and the number of spermatogonia in adult birds in response to increased dietary CP during the growth phase did not affect flock fertility. This may have been because male Japanese quails naturally present high fertility rates (higher than 90%), even with decreased levels of dietary amino acids (Table 6). In addition, the similar sperm concentrations among birds receiving diets with different CP levels indicate that sperm production was regulated by physiological mechanisms [25]. These authors suggest that follicle-stimulating hormone (FSH) secretion is regulated by activin and inhibin. In males, FSH controls inhibin secretion through unknown mechanisms. This hypothesis of physiological regulation can be reinforced by the fact that increases in the number of Sertoli cells and spermatogonia were not associated with increases in the sperm concentration of the birds (Table 5).

During the growth phase, rapid body development is directly related to reproductive organ development [26]. Thus, the supply of amino acids during this phase may affect bird growth. In the present study, an increase of up to 24% in dietary CP accelerated body development and led to earlier maturity of growth. This rapid development resulted in increased testicular size and gonadosomatic index at the end of the growth phase (35 days of age), but not at 60 days of age. The reduction in Leydig cell concentration per testicular unit observed with increased CP in the diet suggests that testicular enlargement is not related to the number of Leydig cells but only to the number of tubular cells (spermatogonia and testis). This hypothesis is reinforced by the increase in the tubule: intertubule ratio stimulated by the increase in dietary CP. This could be related to an earlier reproductive age in males. In females, the increased body weight resulting from increased dietary CP also resulted in faster development of sex organs, decreasing the age of onset of sexual activity [2]. Therefore, using similar diets (with 24% CP) for males and females during the growth phase may benefit the reproductive system because it may lower the age of onset of reproductive activity. In addition, using similar diets for both males and females provides a greater ease of bird handling.

Overall, the amino acid supply should first meet the maintenance requirements, followed by the body development and reproductive activity requirements [1]. In birds, excess amino acids are deaminated, and excess nitrogen is mainly eliminated as uric acid [27]. In Japanese quails, bodily differences between males and females are evident only from 35 days of age when the production phase begins [4,23]. However, growth decelerates (age of maximum growth) in males at approximately 13 days of age, whereas for females, it decelerates at approximately 23 days of age on diets containing 24% CP, as recommended by the National Research Council (NRC [4]). In this study, growth decelerated between 13 and 14 days of age on diets with CP levels between 22% and 26%. These results indicate that phased nutritional management during bird development may be more effective. Rostagno et al [23] recommended that nutrition in Japanese quails be managed in two phases: chicks (1 to 14 days) and growers (15 to 35 days). Because males present lower growth rates than females, especially from 15 to 35 days of age, these nutritional requirements are likely overestimated for males. This explains the increased nitrogen excretion resulting from increased dietary CP intake (Table 4). The absence of significant differences in nitrogen retention indicates that the amino acid supply in diets with 18% CP met the birds’ maintenance and growth requirements at the end of the growth phase. Live weight gain during the last week of growth (27 to 35 days of age) was similar among the groups with different dietary CP levels (13 to 15 grams).

In this study, the live weights of 15-day-old males increased linearly with increasing dietary CP, reaching the optimal point at 23.9% CP (Figure 4). This is consistent with the performance data for this phase (1 to 14 days), which showed that diets with 24% and 26% CP resulted in more bird weight gained and lower feed conversion (Table 4). In this case, diets with lower CP levels may have limited bird growth because of their lower amino acid supplies. Karaalp [28] suggested that all essential amino acid levels are sufficient in the 24% CP corn- and soybean meal-based diets recommended for Japanese quails in the growth phase by the NRC [4].

The protein requirements for bird body weight gain decreased until 36 days of age, decreasing from 24% over the first two weeks to 20% at the end of the growth phase (Figure 4), which was also observed by Karaalp [28] and Wen et al [22]. In addition, weight gain at the end of the growth phase (1 to 35 days) did not differ among birds receiving diets with 22%, 24%, or 26% CP. This indicates that phased feeding is ideal for quails during the growth phase [23,28] because dietary manipulation may be important in decreasing the nitrogen content in the excreta to ameliorate environmental emissions and respiratory problems in sheds [29]. The current recommendations proposed by Rostagno et al [23] are similar to the results presented here.

Improved knowledge of CP requirements for different animal species and categories through applying the concept of dietary formulations with ideal protein composition to optimize amino acid use is also needed. A decrease of greater than three or four percent in dietary CP, even if the feed meets all the amino acid requirements, may result in low productive performance [30]. Thus, determining the true dietary CP requirements for male Japanese quails is essential to correctly apply the optimal protein concept to breeder flocks.

In this study, levels of 23% CP or higher accelerated bodily and reproductive organ development during the growth phase, affecting the testis up to reproductive age. However, requirements for the chick and grower phases may differ, and further studies are needed to determine the viability of using specific formulations for each rearing phase. In addition, no need for specific dietary formulations in males was evident in this study.

In conclusion, dietary CP concentration affected body and testicular development in male Japanese quails but did not affect reproductive efficiency.

## Figures and Tables

**Table 1:** Calculated percentage compositions and nutrient levels in Japanese quail feed during the growth (1 to 35 days) and production (36 to 96 days) phases

Variable	Crude protein (%)					

	18	20	22	24	26	Production
Corn	56.30	52.40	48.55	44.70	40.80	53.00
Soybean meal 45%	28.80	33.89	38.95	44.03	49.10	29.43
Wheat meal	2.00	2.00	2.00	2.00	2.00	3.00
Bicalcium phosphate	1.240	1.192	1.145	1.098	1.050	1.06
Calcitic lime	1.35	1.35	1.35	1.35	1.35	6.785
Vegetable oil	3.50	3.58	3.65	3.73	3.80	2.75
Common salt	0.399	0.399	0.398	0.397	0.397	0.322
Mineral supplement^1)^	0.100	0.100	0.100	0.100	0.100	0.100
Vitamin supplement^2)^	0.100	0.100	0.100	0.100	0.100	0.100
DL-methionine (99%)	0.005	0.038	0.070	0.103	0.136	0.604
L-lysine (78%)	0.052	0.039	0.026	0.013	0.000	0.358
L-threonine (99%)	0.002	0.015	0.031	0.046	0.060	0.038
L-tryptophan (98%)	0.004	0.003	0.002	0.001	0.000	0.016
Choline chloride (60%)	0.050	0.050	0.050	0.050	0.050	0.037
Kaolin	6.00	4.75	3.50	2.25	1.00	2.30
Total	100.00	100.00	100.00	100.00	100.00	100.00
Calculated nutritional composition						
Metabolizable energy (kcal/kg)	2,900	2,900	2,900	2,900	2,900	2,800
Crude protein analyzed (%)	18.32	20.09	21.92	24.14	26.39	18.04
Calcium (%)	0.900	0.900	0.900	0.900	0.900	2.909
Available phosphorus (%)	0.333	0.333	0.333	0.333	0.333	0.303
Sodium (%)	0.176	0.176	0.176	0.176	0.176	0.145
Amino acid/lysine						
Lysine (%)	100	100	100	100	100	100
Methionine+cystine (%)	56	56	56	56	56	82
Threonine (%)	69	69	69	69	69	60
Tryptophan (%)	23	23	23	23	23	21

1) Content per kg of feed (minimum for all elements): 10 mg of copper, 50 mg of iron, 1.2 mg of iodine, 80 mg of manganese, 0.28 mg of selenium and 60 mg of zinc.

2) Content per kg of feed: 0.8 mg of folic acid, 35 mg of pantothenic acid, 1.0 mg of biotin, 40 mg of niacin, 11,500 IU of vitamin A, 3.0 mg of vitamin B_1_, 22 IU of vitamin E, 0.6 mg of vitamin B_12_, 4.4 mg of vitamin B_2_, 10.0 mg of vitamin B_6_, 2,100 UI of vitamin D_3_, 1.5 mg of vitamin K_3_, and 125 mg de antioxidant.

**Table 2:** Growth characteristics obtained from the Gompertz curve for male Japanese quails between 1 and 60 days old receiving diets with different crude protein levels during the growth phase (n = 12)

Variable	Crude protein (%)					SEM	p-value

	18	20	22	24	26		
Body weight at growth maturity (g)	149	147	146	141	141	0.42	0.55
Growth rate	0.057^*^	0.069	0.078	0.089	0.091	0.01	<0.01
Age at maximum growth (d)	25.0^**^	16.0	14.1	12.6	12.7	0.35	0.02

SEM, standard error of the mean.

* Linear effect (p<0.01).

** Linear response plateau (p<0.05).

**Table 3:** Live weight of male Japanese quails receiving diets with different crude protein levels during the growth phase (n = 12)

Age (d)	Crude protein (%)					SEM	p-value		
	
	18	20	22	24	26		Protein	Day	Prot×Day
6	16.3	19.5	22.9	22.7	24.6	3.16	<0.01	<0.01	<0.01
15	40.5^*^	52.2	57.1	63.7	61.6				
21	58.8^*^	74.6	79.7	86.1	87.6				
27	77.2^*^	96.4	100.6	106.1	107.2				
36	91.4^*^	111.1	113.8	119.9	122.0				
42	112.4^**^	124.9	131.7	134.4	130.2				
48	125.9^**^	131.3	136.7	137.6	133.3				
54	132.5	134.7	137.9	135.0	135.1				
60	134.2	135.9	140.4	135.4	136.5				

SEM, standard error of the mean.

* Linear response plateau (p<0.01).

** Quadratic effect (p<0.01).

**Table 4:** Performance and nitrogen balance of male Japanese quails receiving diets with different crude protein levels during the growth phase (n = 6)

Variable	Crude protein (%)					SEM	p-value

	18	20	22	24	26		
1 to 14 days							
Weight gain (g)	24.8^a^	35.2^b^	38.6^b^	46.2^c^	45.6^c^	0.13	<0.01
Feed intake (g)	65.1^a^	86.2^b^	88.6^b^	89.4^b^	94.7^b^	3.78	<0.01
Feed conversion	2.65^a^	2.45^ab^	2.29^b^	1.96^c^	2.07^c^	0.11	<0.01
1 to 35 days							
Weight gain (g)	84^a^	102^b^	108^c^	110^c^	111^c^	0.26	<0.01
Feed intake (g)	289^a^	335^b^	350^b^	353^b^	359^b^	6.68	<0.01
Feed conversion	3.46	3.29	3.25	3.22	3.25	0.06	0.07
Nitrogen balance at 35 days							
N ingested (mg/bird/d)	0.41^*^	0.49	0.54	0.59	0.66	0.01	<0.01
N excreted (mg/bird/d)	0.19^**^	0.25	0.30	0.34	0.40	0.01	<0.01
N retained (mg/bird/d)	0.22	0.23	0.25	0.25	0.26	0.01	0.32

SEM, standard error of the mean.

a–c Means followed by different letters within the same row differ significantly by the Student-Newman-Keuls test (p<0.05).

* Linear effect (p<0.05). y = 0.059x+0.365; R^2^ = 0.99.

** Linear effect (p<0.05). y = 0.049x+0.153; R^2^ = 0.98.

**Table 5:** Growth characteristics and histological analyses of the testes of male Japanese quails aged 35 (n = 12) and 60 (n = 6) days receiving diets with different crude protein levels during the growth phase

Variable	Crude protein (%)					SEM	p-value

	18	20	22	24	26		
	------------------------------------------------- 35 days ----------------------------------------------						
Gonadosomatic index	0.53^*^	0.45	0.98	1.38	1.53	0.11	<0.01
Right testis							
Weight (g)	0.24^*^	0.24	0.52	0.76	0.80	0.05	<0.01
Height (mm)	8.27^*^	9.60	13.33	14.79	14.99	0.85	<0.01
Width (mm)	5.06^*^	5.65	7.58	8.58	9.05	0.52	<0.01
Thickness (mm)	4.88^*^	5.41	7.08	8.30	8.58	0.48	<0.01
Volume (cm^3^)	0.11 ^*^	0.20	0.41	0.60	0.66	0.06	<0.01
Left testis							
Weight (g)	0.24^*^	0.24	0.56	0.76	0.94	0.06	<0.01
Height (mm)	7.70^*^	8.88	12.54	13.62	14.13	0.78	<0.01
Width (mm)	5.56^*^	6.03	8.44	9.44	10.35	0.61	<0.01
Thickness (mm)	5.50^*^	5.87	8.15	9.02	9.85	0.59	<0.01
Volume (cm^3^)	0.13^*^	0.21	0.50	0.66	0.82	0.06	<0.01
Seminiferous tubular area (μm^2^)	13,314^*^	15,806	28,986	33,727	34,927	3,289	<0.01
Germinal epithelial height (μm)	42.6^**^	46.7	63.0	63.6	65.8	4.18	<0.01
Tubule:intertubule ratio	11.3^a^	17.2^b^	17.9^b^	21.1^c^	20.6^c^	2.60	<0.01
Leydig cells/area (10.000/μm^2^)	10.7^*^	10.0	7.3	5.7	5.1	0.57	<0.01
Leydig cells/intertubule (%)	36.9^*^	34.4	25.0	19.5	18.9	0.34	<0.01
Sertoli cells/area (10.000/μm^2^)	2.47	2.44	2.60	2.21	2.45	0.34	0.95
Sertoli cells/tubule	22.6	19.1	19.2	15.6	16.5	1.84	0.10
Spermatogonia/area (10.000/μm^2^)	3.69^*^	4.34	7.88	8.41	9.55	0.80	<0.01
Spermatogonia/tubule	27.1^*^	33.0	54.8	62.0	63.7	0.31	<0.01
% proliferating spermatogonia	74.7^*^	83.1	88.4	95.9	97.2	0.34	0.01
	------------------------------------------------- 60 days ---------------------------------------------						
Gonadosomatic index	4.39	4.03	3.67	4.01	3.87	0.17	0.15
Right testis							
Weight (g)	2.73	2.51	2.61	2.49	2.41	0.08	0.15
Height (mm)	23.05	23.63	23.3	22.98	22.41	0.56	0.75
Width (mm)	14.27	13.96	13.81	13.8	13.53	0.30	0.66
Thickness (mm)	13.58	13.00	13.29	13.27	12.85	0.18	0.16
Volume (cm^3^)	2.36	2.25	2.09	2.21	2.06	0.12	0.37
Left testis							
Weight (g)	2.86	2.54	2.7	2.57	2.66	0.03	0.54
Height (mm)	21.67	21.17	20.67	20.73	21.39	1.09	0.59
Width (mm)	15.08	14.72	15.24	14.88	14.92	0.31	0.87
Thickness (mm)	14.77	14.31	14.55	14.48	14.49	0.33	0.94
Volume (cm^3^)	2.55	2.35	2.42	2.36	2.43	0.15	0.89
Seminiferous tubular area (μm^2^)	73,477	75,312	69,823	72,781	67,841	3,576	0.60
Germinal epithelial height (μm)	86.9	89.4	87.9	88.5	84.1	2.82	0.72
Tubule:intertubule ratio	33.1	32.9	42.9	33.6	41.5	6.13	0.63
Leydig cells/area (10.000 μm^2^)	24.8^a^	14.4^b^	13.1^b^	11.5^b^	11.8^b^	3.12	<0.01
Leydig cells/intertubule (%)	13.6^a^	7.9^b^	7.0^b^	6.3^b^	6.5^b^	1.71	<0.01
Sertoli cells/area (10.000 μm^2^)	1.16^*^	1.23	1.32	1.43	1.58	0.05	<0.01
Sertoli cells/tubule	8.25^*^	8.51	9.66	10.28	10.67	0.47	<0.01
Spermatogonia/area (10.000 μm^2^)	10.9^a^	10.3^a^	10.9^a^	11.4^b^	12.7^c^	0.55	0.05
Spermatogonia/tubule	80.1^a^	78.3^a^	76.8^a^	84.0^b^	84.7^b^	3.95	0.05
% proliferating spermatogonia	95.5	95.3	96.1	94.1	94.9	0.65	0.31

SEM, standard error of the mean.

* Linear effect (p<0.01).

** Linear response plateau (p<0.01).

a,b Means followed by different letters within the same row differ significantly by the Student-Newman-Keuls test (p<0.05).

**Table 6:** Reproductive performance of male Japanese quails aged 90 days receiving diets with different crude protein levels during the growth phase (n = 6)

Variable	Crude protein (%)					SEM	p-value

	18	20	22	24	26		
Cloacal gland	13.6	13.9	14.1	14.2	14.1	0.24	0.39
Height (mm)	20.1	20.8	20.4	20.5	21.2	0.40	0.35
Width (mm)	2.73	2.90	2.86	2.91	3.00	0.10	0.38
Area (cm^2^)	0.14	0.16	0.14	0.12	0.11	0.07^*^	0.67
Foam weight (g)	1.62	1.65	1.69	1.71	1.55	0.12	0.87
Foam protein (g/dL)	3.56	3.28	2.85	3.63	3.30	0.38	0.64
Semen volume (mL)	13.6	13.9	14.1	14.2	14.1	0.24	0.39
Semen concentration (×10^6^ sptz/μL)	1.41	1.43	1.30	1.45	1.68	0.20	0.75
Semen without foam							
Motility (%)	67.9	67.1	64.1	70.4	68.9	3.60	0.79
Vigor	3.14	3.07	3.03	3.35	3.06	0.15	0.59
Viability (%)	95.6	95.4	95.6	93.9	96.8	0.42	0.42
Semen with foam							
Motility (%)	68.8	67.9	64.4	67.4	68.4	4.15	0.95
Vigor	3.42	3.58	3.63	3.42	3.79	0.15	0.40
Viability (%)	95.7	86.1	96.3	94.3	95.3	0.44	0.84
Fertility (%)^**^	91.7	89.8	94.4	95.4	90.7	-	0.56

SEM, standard error of the mean.

* Box-Cox data transformation.

** n = 100.
